# Dissection of Maize Drought Tolerance at the Flowering Stage Using Genome-Wide Association Studies

**DOI:** 10.3390/genes13040564

**Published:** 2022-03-23

**Authors:** Siffat Ullah Khan, Yanxiao Zheng, Zaid Chachar, Xuhuan Zhang, Guyi Zhou, Na Zong, Pengfei Leng, Jun Zhao

**Affiliations:** 1 Crop Functional Genome Research Center, Biotechnology Research Institute, Chinese Academy of Agricultural Sciences, Beijing 100081, China; sifat.phd@gmail.com (S.U.K.); zyx080507@163.com (Y.Z.); zs.chachar@gmail.com (Z.C.); zhangxuhuan01@163.com (X.Z.); zhouguyi0805@163.com (G.Z.); zongna@caas.cn (N.Z.); 2 National Nanfan Research Institute (Sanya), Chinese Academy of Agricultural Sciences, Sanya 572024, China

**Keywords:** maize, drought tolerance, flowering stage, GWAS

## Abstract

Drought is one of the most critical environmental factors constraining maize production. When it occurs at the flowering stage, serious yield losses are caused, and often, the damage is irretrievable. In this study, anthesis to silk interval (ASI), plant height (PH), and ear biomass at the silking date (EBM) of 279 inbred lines were studied under both water-stress (WS) and well-water (WW) field conditions, for three consecutive years. Averagely, ASI was extended by 25.96%, EBM was decreased by 17.54%, and the PH was reduced by 12.47% under drought stress. Genome-wide association studies were carried out using phenotypic values under WS, WW, and drought-tolerance index (WS-WW or WS/WW) and applying a mixed linear model that controls both population structure and relative kinship. In total, 71, 159, and 21 SNPs, located in 32, 59, and 12 genes, were significantly (*P* < 10^−5^) associated with ASI, EBM, and PH, respectively. Only a few overlapped candidate genes were found to be associated with the same drought-related traits under different environments, for example, ARABIDILLO 1, glycoprotein, Tic22-like, and zinc-finger family protein for ASI; 26S proteasome non-ATPase and pyridoxal phosphate transferase for EBM; 11-ß-hydroxysteroid dehydrogenase, uncharacterised, Leu-rich repeat protein kinase, and SF16 protein for PH. Furthermore, most candidate genes were revealed to be drought-responsive in an association panel. Meanwhile, the favourable alleles/key variations were identified with a haplotype analysis. These candidate genes and their key variations provide insight into the genetic basis of drought tolerance, especially for the female inflorescence, and will facilitate drought-tolerant maize breeding.

## 1. Introduction

Maize (*Zea mays* L.) is the most widely grown crop worldwide and has incredible importance for food, feed, and other industrial products [1]. It was estimated that maize production needs to be boosted by 67% to cope with the increased population growth and food demand in 2050 [2]. Thus, it is of great significance to increase maize yield productivity and reduce yield loss caused by biotic and abiotic stresses.

Drought is considered one of the most detrimental curbs of agriculture, which endangers maize production globally because of its erratic nature [3,4]. Maize is an open-pollinating crop that is extremely sensitive to drought stress throughout its life span, especially one week before and three weeks after the flowering period, causing severe yield loss by 15–25% [5]. At this stage, drought inhibits ear development, causing abnormal differentiation in spikelets, and changes in hormone signalling involved in cell division, growth, and primordium development [6], leading to asynchronous development in tassel and ear, a tremendous extension of anthesis and silking interval (ASI), and reduction in silk receptivity [7,8]. Consequently, this time slack between pollen release and silk emergence adversely affects pollination and kernel set and results in the reduction in grain yield [9,10]. Drought during the grain filling stage resulted in starch quality and quantity reduction, leading to a great loss of grain weight and grain quality [11]. Drought stress also reduces plant and ear height and decreases the availability of photosynthate for grain production, ultimately leading to yield reduction [12,13]. Due to global warming and increasing water resources shortage, the water deficit has become more and more prominent. Breeding maize for drought tolerance, especially at the flowering stage, is thus of significant importance for global food production.

Association analysis based on linkage disequilibrium (LD) acquired increasing popularity and accuracy in the genetic architecture of polygenic traits in crops due to broader genetic variation, larger numbers of alleles, and the maximum number of recombinants obtained [14,15]. Genome-wide association study (GWAS), taking advantage of natural variation and historical recombination, have emerged as alternative tools to linkage mapping for identifying superior alleles for complex traits, with reduced time consumption and increased mapping resolution [16,17]. GWAS have been employed to identify numerous SNPs directly associated with drought tolerance, especially at the seedling stage. *ZmVPP1* was associated with dehydration tolerance in maize seedlings, encoding a vacuolar-type H^+^ pyrophosphatase, which improved seedling drought tolerance in maize due to enhanced photosynthetic activity and root development [18]. Significant associations were detected between maize seedling drought tolerance and functional genes promoter variation, such as SNPs located in the promoter of *ZmDREB2.7* and miniature inverted-repeat transposable element in *ZmNAC111* promoter, which determined gene expression for dehydration tolerance in maize seedlings [19,20]. A 368 maize association panel was used to conduct an association analysis on *ZmPP2C-A* family genes and found that *ZmPP2C-A-10* was closely related to drought stress, through regulating the ABA signalling pathway [21]. This study reveals the correlation between endoplasmic reticulum stress response and drought resistance.

Research on maize drought-tolerant genes for the flowering period is scarce and relatively lagging, which is likely because maize drought tolerance is a set of traits with trade-offs, and plasticity and the internal connections are extremely complicated. However, few genes contributing to drought tolerance in maize were identified through GWAS or linkage mapping using a larger-scale population due to uncontrollable field conditions. NAC transcription factor NUT1 was found specifically expressed in protoxylem at the flowering stage, and functions to manipulate water transport by maintaining protoxylem vessel integrity through activating genes necessary for secondary cell wall reinforcement, thus affecting drought tolerance in NUT1 mutants [22]. A recent study found that *ZmEXPA4*, identified through transcriptomics analysis, functions during ear growth and silk elongation, alleviating drought-induced ASI elongation without affecting other agronomic traits [23]. More recently, using a high-throughput phenotyping platform, 368 maize inbred lines were continuously non-destructive, tested under normal watering and drought stress conditions at multiple growth stages, resulting in 2318 candidate genes associated with i-traits and drought tolerance in maize [6]. Mutant-based functional validation has shown that *ZmcPGM2* (involved in sugar metabolism) and *ZmFAB1A* (involved in phosphoinositide metabolism) can negatively regulate drought resistance in maize during flowering.

In this study, an association population consisting of 279 inbred lines was evaluated under field WW and WS conditions. We aimed to dissect the drought-tolerant candidate genes at the flowering period through GWAS and to provide potential genetic variations for breeding drought-tolerant maize varieties.

## 2. Materials and Methods

### 2.1. Plant Materials and Experimental Design

An association panel for GWAS comprising 279 inbred lines with diverse genetic variation (Table S1) was selected from comprehensive geographical regions including tropical, subtropical, and temperate areas of the world, as described by Zhang et al. [24]. This panel was obtained from an available association population for eQTL mapping on maize kernel development [25,26].

The field trials were conducted in Urumqi (Xinjiang province: 43°54′ N, 87°28′ E), from May to September. The flowering date of the association panel was recorded in 2016 under normal growing conditions and then divided into three patches according to growing degree day (GDD) value at −15 days before pollen shedding (−15 D). The daily temperature was recorded, and GDD was calculated for each line using the following formula:

$$GDD=\frac{\left(L+H\right)}{5}-50$$

where *L* means daily lowest temperature (°F), and *H* means daily highest temperature (°F).

**Figure 1:**
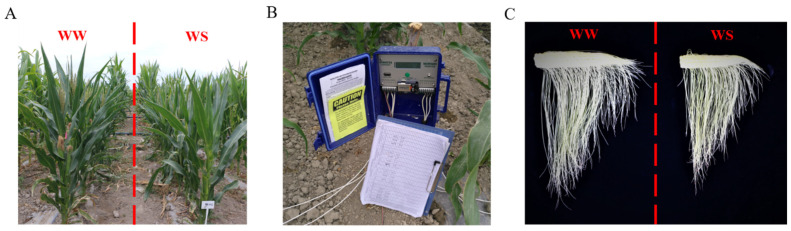
Soil moisture monitor and plant performance under different water treatment: (**A**) WATERMARK MONITOR installed in the field and connected to the sensor; (**B**,**C**) plant height and ear development of drought-sensitive lines under WW and WS condition.

In order to obtain synchronised flowering, 279 lines were delayed sowing in patches. Each patch was in randomised complete block design, with three independent repeats during the years of 2017, 2018, and 2020. A total of 119 lines belonged to the 1st patch (No. 1–119, Table S1), with GDD ranging from 1612.1 to 1901.9 for the −15 D, and were planted on 8 May 2017. A total of 138 lines were planted 10 days later than those of the first patch (18 May 2017), with GDD values at −15 D ranging from 1324.7 to 1599.3. The remaining 22 lines (No. 258–279, Table S1) with earlier flowering times (GDD = 962–1291.6) were planted on 28 May 2017. Each row was 3.6 m in length, with planting space of 0.24 m and 1.1 m width across rows. Every line was planted side by side, one row for water stress treatment (WS) and the adjacent for a well-watered regime (WW, Figure 1A), with an independent valve to control the irrigation of each row. Drought stress was applied in the WS regime under the Protocol of Irrigation Management for Maize Drought Trials of Syngenta. Briefly, daily moisture level was recorded using the soil moisture sensors WATERMARK MONITOR 900 M (IRROMETER) installed in the field (Figure 1B) and connected to the sensor in every patch at 60 cm and 90 cm depth in multiple locations according to instructions from the trial sponsor in both WS and WW regimes. For the intended drought stress management at the flowering stage, a drought stress trial was conducted by withdrawing irrigation initiated at the −21 D before anthesis estimated according to GDD. The soil moisture was maintained at <40 centibars before −15 D and at 80–120 centibars from −15 D to −7 D. At −7 D, the soil moisture reached 120–150 centibars, which is the trigger point for drought stress, and was kept at 150–200 centibars until 14 days after anthesis when irrigation was resumed. The fertilisers, herbicides, and insecticides were applied as per requirements according to the local recommendation practices. In the WW regime, normal irrigation was provided once a week.

### 2.2. Phenotyping for Drought-Stress-Related Traits

PH and flowering time, such as anthesis date and silking date, were examined in inbred lines under WS and WW conditions. Days to anthesis (AD) and days to silking (SD) were determined by the number of days from planting to pollen shedding of 50% of plants and 50% of plants having clearly visible silks, respectively. ASI was calculated as the interval between anthesis date and silking date (ASI = SD − AD) and counted in days [9]. PH was measured from the soil level to the lowest tassel branch of each plant and recorded in centimetres (cm). For ear biomass, the ear at its silking date (Figure 1C) of each line was harvested and dried to a consistent weight at 72 °C. The ear biomasses were weighed with an electronic weighing balance and recorded in grams (g). The drought-tolerant index was calculated by dividing the mean values of studied traits in the WS regime by those of the WW regime. To estimate the random errors, each measurement per inbred line comprised 6–8 individual plants with 3 independent repeats.

### 2.3. Association Analysis for ASI, EBM, and PH

For association analysis, the 279 lines were genotyped by 776,254 high-density SNP markers, with MAF > 0.05, which was excavated previously [26]. The genome-wide association analysis was conducted with a mixed linear model (MLM) embedded in TASSEL V5.0 [27]. Population structure (Q) and kinship (K) were estimated according to Pang et al. [26]. Briefly, kinship matrices and principal components were estimated based on 236,205 SNPs with MAF > 0.05. The top 3 principal components were selected as population structure, and for kinship estimation, the ‘Normalised_IBS’ method was used. The regression-based coefficient of determination values of all significantly associated SNPs was recorded to determine the variations explained by each SNP locus. Drought tolerance was controlled by several genes with minor effects and non-independence of SNPs in maize genome leading to strong LD. Therefore, a threshold of *P* < 10^−5^ was applied in this study to eliminate false negatives. Multiple Manhattan plots were drawn in R (https://www.r-project.org/ (accessed on 8 November 2021)).

Based on the maize B73 reference genome (V4), genes directly colocalised with significantly associated SNPs considered as candidates. Alternatively, genes located within a corresponding LD interval (*r*^2^ ≥ 0.2) were also considered [18]. The gene annotation and function were retrieved from the Maize Genomics database (http://www.maizegdb.org/ (accessed on 6 December 2020)), Gramene database (http://gramene.org/ (accessed on 6 December 2020)), NCBI (https://www.ncbi.nlm.nih.gov/ (accessed on 6 December 2020)), and other available sources in the literature.

### 2.4. Drought Responsive, Linkage Disequilibrium, and Haplotype Analysis of Candidate Genes

In total, 197 inbred lines from a previous association mapping population were planted in pots under well-watered and drought stress treatments [6]. Drought stress conditions were relative to WW conditions, with the soil moisture levels of WS conditions dropping from 50% to 10% and soil moisture levels of WW conditions keeping values around 50% at the flowering stage. Candidate genes—*Zm00001d013992*, *Zm00001d020506*, *Zm00001d029937*, *Zm00001d029938*, and *Zm00001d039319*—were commonly identified under two environments. Therefore, to determine whether they were drought-responsive, their expression patterns were analysed according to the RNA profiling data of the abovementioned 197 lines (unpublished data from Dr. Mingqiu Dai, Huazhong Agricultural University). Haplotype analysis normally helps to understand the causal variation in a gene identified by GWAS [20,26], which in turn benefits future functional marker development. To confirm whether the SNPs identified for each gene were meaningful, haplotype analysis was conducted to check the allele effects of the most significant SNPs for the six overlapping candidate genes. A two-pair *t*-test was used to analyse their allelic effect in corresponding phenotypic performance.

### 2.5. Statistical Analysis

The phenotypic data were filtered by removing the suspicious value of each replicate by *Q-test* in excel [24]. The mean value of three replicates in each environment was used for association analysis. Best linear unbiased estimators (BLUEs) were estimated using the genotype and covariate as a fixed factor, and the rest as random factors. Analysis of variance (ANOVA), correlations analysis, and broad-sense heritability (*H*^2^) were estimated by SPSS 25.0. The *H*^2^ was estimated according to the following equation:

$$H^{2}=\frac{\sigma_{G}^{2}}{\sigma_{G}^{2}+\sigma_{GE}^{2}/n+\sigma_{e}^{2}/nr}$$

where *σ*^2^*_G_* represents genetic variance, *σ*^2^*_GE_* shows the genetic and environmental interaction variation, *σ*^2^*_e_* shows residual error variance, *n* shows the number of environments, and *r* shows the number of replicates [28]. LD analysis of these candidate genes was conducted by SNPs within a gene using the ‘LD heatmap’ in the R software (https://www.r-project.org/ (accessed on 8 November 2021)).

## 3. Results

### 3.1. Performance of Drought-Tolerant Phenotypes in the Association Panel

**Figure 2:**
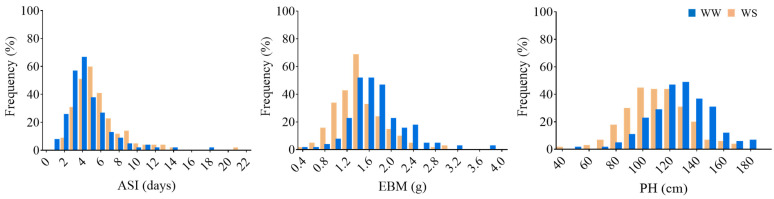
Frequency distributions of ASI, EBM, and PH under two water treatments.

The test of normality revealed that the frequency distributions for all of the studied traits were near normal for most of the traits in the association panel (Figure 2; Table 1). The existence of variation might be caused by fluctuating environmental conditions in the field or genotypic differences. The descriptive statistics, heritability analysis, and coefficient of variation (CV) for the phenotypic traits are listed in Table 1. Generally, drought stress significantly decreased the ear biomass and PH (Figure 1B,C) while enlarging the ASI, suggesting that water stress at the flowering stage had diverse effects on drought-related traits.

The mean values of ASI under drought stress were 5.58 days with the CV 42.97%, while under WW condition, ASI was 4.43 days, with a CV value of 48.89% (Table 1). The average ASI across the three years under the WS regime was 1.15 days larger than that of the WW regime. Under drought stress, ASI was increased by 25.96%, indicating that drought stress significantly enlarged silk extrusion at the population level. Drought stress caused a 17.54% reduction in EBM with a minimum of 0.48 g and a maximum of 3.08 g under WS conditions, while they were 0.48 g and 3.81 g under the well-watered regime. The mean value of ear biomass was 1.71 g under the WW condition, with a CV of 27.20%, while it was 1.41 g under the WS condition, with a CV of 29.15% for the three-year environments, respectively (Table 1), suggesting a suppressed ear development by drought stress. Likewise, drought stress caused a 12.47% reduction in PH; the minimum PH was 43.21 cm, and the maximum was 169.72 cm under the WS condition, while they were 51.23 cm and 183.74 cm under the WW condition. The mean value of PH under the WS regime was 111.36 cm with a CV of 19.34%, significantly lower than that in the WW regime, which was 127.22 cm with a CV of 16.92% for the three years, respectively (Table 1). *H*^2^ values of ASI, EBM, and PH were higher than 80% under the two water treatments across the three years (Table 1), indicating that these traits are highly heritable in the two water treatment conditions.

### 3.2. Correlations among Drought-Related Traits

The correlations among ASI, EBM, and PH values under WS and WW conditions are listed in Table 2. Significantly positive correlations (*P* < 0.05) were found between ASI and PH under WS (0.13) and WW (0.15) conditions. Additionally, significant correlations were found between ear biomass and PH under both WS (0.22) and WW (0.18) conditions. A weak and negative correlation was found between ASI and ear biomass.

### 3.3. GWAS for Maize Drought Tolerance Genes

**Figure 3:**
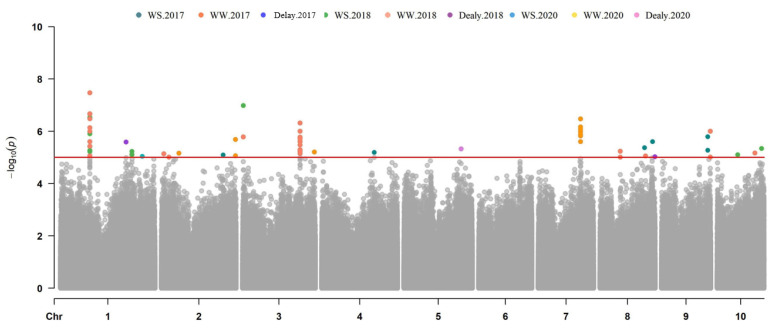
Manhattan plots of ASI using MLM model in three years.

**Figure 4:**
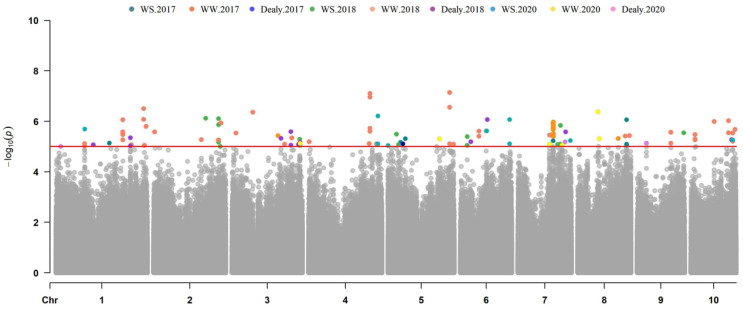
Manhattan plots of ear biomass using MLM model in three years.

**Figure 5:**
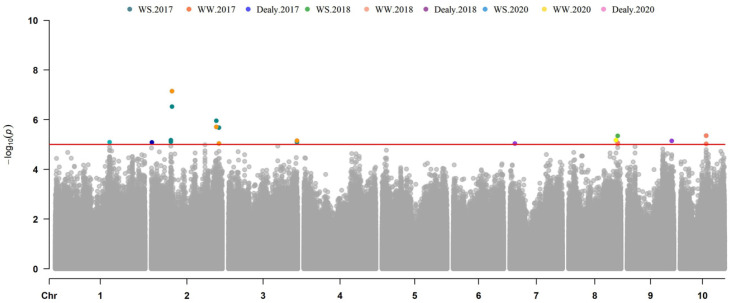
Manhattan plots of plant height using MLM model in three years.

A total of 71, 159, and 21 SNPs were significantly associated with ASI, EBM, and PH located in 36, 81, and 16 genes, respectively. These genes were scattered over 10 chromosomes, with *R*^2^ ranging from 7.19% to 15.52%. The number of identified SNPs in each chromosome ranged from 9 to 31 on chromosomes 9 and 1, respectively. The information about the SNPs and candidate genes is shown in Tables S2–S4. A total of 17 and 48 SNPs located in 13 and 18 genes were identified for ASI-WS and ASI-WW, with *R*^2^ ranging from 7.73% to 14.32% (Figure 3; Table 3 and Table S2). As to the ASI delay, only five SNPs in four genes were identified in 2017 and 2018 (Table S2). In total, 49 and 93 SNPs located in 23 and 43 were associated with EBM-WS and EBM-WW, with *R*^2^ ranging from 7.99% to 12.52% (Figure 4; Table 3 and Table S3). In addition, 17 SNPs in 15 genes were associated with the EBM drought tolerance index (WS/WW) across the three years (Table S3). For both conditions, nine SNPs were associated with PH-WS and PH-WW and located in seven and six genes, with *R*^2^ ranging from 7.19% to 15.52% across the three-year environments (Figure 5; Table 3 and Table S4). Three genes in Chr. 2, 7, and 9 were identified for the PH drought tolerance index (Table S4).

### 3.4. Common Genes Identified for Ear Development across Multiple Years or Conditions

Several overlapping genes were identified among years and water treatments (Table 3). Four candidate genes encoding ARABIDILLO 1 protein (Zm00001d029938) and Glycoprotein (Zm00001d029937) on Chr. 1, Tic22-like family protein (Zm00001d039319), and zinc-finger family protein (Zm00001d042997) on Chr. 3, were identified for ASI under both WS and WW conditions in 2018 (Figure 3), suggesting that these genes might be promising candidates, as they can function in maize inflorescence development with or without water deficit. An SNP (S5_27121944), located in pyridoxal phosphate transferase encoding gene (*Zm00001d013992*), was significantly associated (*P* < 10^−5^) with EBM under drought stress condition on Chr. 5 and was consistently detected for two years environments of 2018 and 2020 (Figure 4), suggesting the drought tolerance role of this gene in developing ear. A candidate gene (*Zm00001d020506*) encoding 26S proteasome non-ATPase regulatory subunit-9 was detected for EBM on Chr. 7 under both WS and WW regimes during the 2017 field trial (Figure 4), suggesting its possible role in maize ear development.

### 3.5. Candidate Genes Drought Responsive Pattern

**Figure 6:**
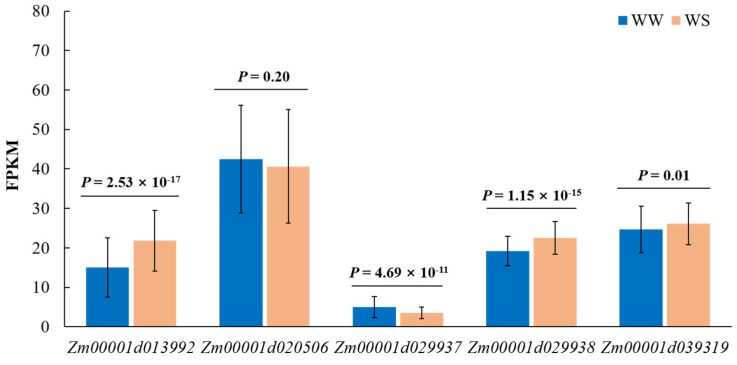
Drought responsive pattern of candidate genes under WS and WW conditions.

Most quantitative traits’ functional genes are responsive at the transcriptional level. Drought tolerance is complex and regulated by many quantitative trait loci (QTLs) with minor effects. In order to determine whether these candidate genes were drought-regulated, we analysed their expression level using the expression data from 197 diverse inbred lines under WS and WW conditions. Unsurprisingly, significant differential expression existed in most candidate genes between WW and WS treatments. *Zm00001d013992*, *Zm00001d029938*, and *Zm00001d039319* were increased by 45.44%, 17.46%, and 6.01%, while *Zm00001d029937* was reduced by 30.26%, compared with their expression under the WW condition (Figure 6). However, no obvious difference was observed for *Zm00001d039319*.

### 3.6. Allele Effects of Common Candidate Genes

**Figure 7:**
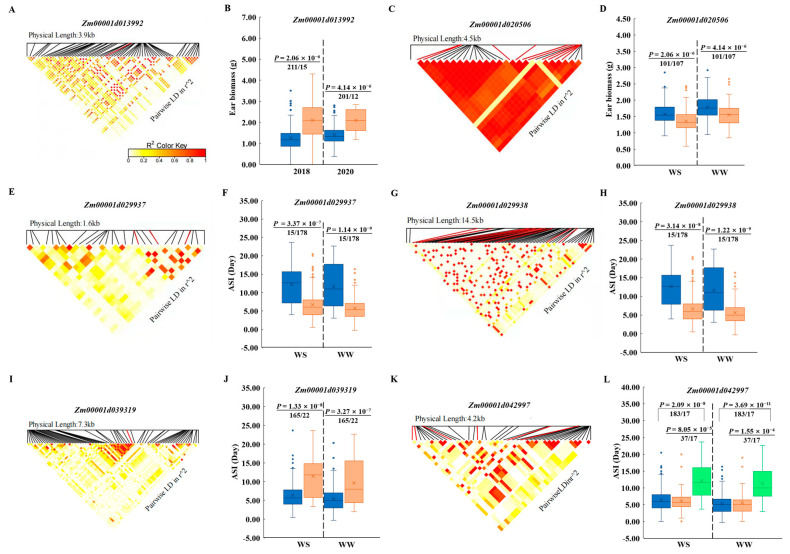
LD patterns and the allele effects of the most significant SNPs for candidate genes *Zm00001d013992* (**A**), *Zm00001d020506* (**C**), *Zm00001d029937* (**E**), *Zm00001d029938* (**G**), *Zm00001d039319* (**I**), and *Zm00001d042997* (**K**). Results of haplotype analysis for *Zm00001d013992* (**B**), *Zm00001d020506* (**D**), *Zm00001d029937* (**F**), *Zm00001d029938* (**H**), *Zm00001d039319* (**J**), and *Zm00001d042997* (**L**). Blue: *HapA*; pink: *HapB*; green: *HapC*.

SNPs within a gene might be critical to its function. Therefore, it was of great interest to find the true association between SNP variation and target traits in a population. Haplotype analysis is a useful strategy to extract more rare causal variants. In our study, inbred lines were grouped into *HapA* (A) and *HapB* (G) based on the most significant SNP (S5_27121944) variation in *Zm00001d013992* (Table 3, Figure 7A), 226 (2018), and 213 (2020). The rare *HapB* group had significantly higher EBM (2.10 g) under WS conditions in both 2018 and 2020 (Figure 7B, Table S5) than *HapA*. Five polymorphisms—namely, chr7.S_116288756, chr7.S_116288791, chr7.S_116288792, chr7.S_116285652, and chr7.S_116285655—were identified in *Zm00001d020506* (Figure 7C, Table S6). In total, 101 lines belonged to the *HapA* (AACCT) with average EBM values of 1.58 g and 1.79 g under WS and WW in 2017, while the other 107 lines carried the GTTTC haplotype with significantly lower EBM values of 1.35 g and 1.55 g under WS and WW (Figure 7D). Three SNPs in *Zm00001d029937* were significantly associated with ASI under the WW condition in 2018 (Table 3, Figure 7E), and accordingly, two haplotype AAC (*HapA*) and GGG (*HapB*) were obtained. Of the 193 lines harbouring rare haplotype *HapA*, 15 showed longer ASI (>10 days) than those harbouring *HapB*, whose ASI values were 5.6 days and 6.6 days under WW and WS conditions, respectively (Figure 7F, Table S7). The same 15 lines belonging to the *HapA* group (TCGATAATC) presented longer ASI under both WW and WS conditions (Figure 7G,H, Table S8), which was a similar case as that of the adjacent gene *Zm00001d029938*. PZE-103003226 was the only SNP identified in *Zm00001d039319* (Figure 7I). The 165 lines with shorter ASI, grouped into *HapA* (G), showed weaker sensitivity to drought stress in both WW and WS conditions (Figure 7J, Table S9). For *Zm00001d042997*, 9 SNPs classified the 237 inbred lines into 3 groups (Figure 7K, Table S10), among which 17 lines belonged to lower frequency of *HapC* (TGACTTAA) and had a larger ASI under both WS and WW conditions, compared with those belonging to *HapA* (AACTCCCG) and *HapB* (AACTCTAA), while no statistical difference was found between *HapA* and *HapB* (Figure 7L).

## 4. Discussion

Maize is highly sensitive to drought stress at the flowering stage. In this study, the silk extrusion was markedly delayed, and plant architecture was greatly affected under drought stress. These results are in line with previous reports that stated significant extension in ASI and a notable reduction in plant and ear height under WS conditions [29,30]. Water deficit at the flowering stage delays or inhibits plant growth and ear development, reducing ear biomass from 1.71 g to 1.41 g (Table 1). It was found that osmotic stress limits the dry matter accumulation by approximately 50% during serious water shortages [31,32]. Thus, considering the higher estimated heritability and vulnerability to drought stress, ear biomass could be an optimised option for improving maize selection under water-scarce conditions [33,34]. Plant height was significantly correlated with yield-related traits under both WW and WS conditions [35]. The average PH decreased less when the plant was exposed to drought, which ensures sufficient ‘source’ availability and exhibits better drought resistance. PH was positively correlated with EBM, suggesting the meaningful role of EBM in drought tolerance. A weak and negative correlation existed between the ASI and ear biomass, indicating that delayed silk extrusion has likely no correlation with ear development. Therefore, the three traits may not be tightly correlated with each other, or the correlation may be disturbed due to the variation of the fluctuating environmental conditions in the field.

Up to now, only few GWAS have been conducted on maize drought tolerance under complex field conditions [30,35]. Researchers tested a number of maize-nested association mapping populations under two contrasting water regimes for seven drought-related traits, including ASI-, PH-, and yield-related traits. Hundreds of promising QTLs and candidate genes were obtained through GWAS and linkage mapping [35]. In addition, many other candidate genes were detected to be associated with drought-tolerance-correlated yield and agronomic traits [30,36,37,38]; however, none of them were found in this study, which may be due to the various growth conditions and drought treatments. Initially, we aimed to find candidate genes contributing to drought tolerance, especially those for the drought tolerance index. However, only a few genes were identified, and no overlapping genes were identified across different environments, which might be due to the inherence of drought tolerance controlled by multiple minor effect genes. Fortunately, few candidate genes identified in this study were colocalised with reported QTLs. The uncharacterised gene *GRMZM2G173084*, associated with ASI-WW in 2017, overlapped in a QTL for both ASI-WW and ASI-WS, which was detected by joint linkage analysis in a CN-NAM population [35]. *Zm00001d003939*, encoding 11-ß-hydroxysteroid dehydrogenase 2, is the candidate gene for PH-WS-2017 located in a consistent QTL for PH-WW, which was identified in both CN-NAM and US-NAM populations. In addition, several overlapping genes were identified under water treatments among different years, while only *Zm00001d013992* was commonly identified in a two-year environment. This result implied that maize drought tolerance is a complex trait, highly affected by environment and treatment. Hu et al. [39] reported that grain yield in WS (*qWS-GY7-1*) and an ear setting percentage in WW (*qWW-ESP7-1*), located in Chr. 7: 132.2–135.6 Mb, and seven other QTLs and one mQTL for drought-related traits also clustered in this region [40,41]. Coincidentally, *GRMZM2G173084* encoding an uncharacterised protein was associated with ASI-WW in 2017. Additionally, Hu et al. [39] found that the drought tolerance allele of Chr. 7: 132.2–135.6 Mb improved GY in both WW and WS regions, suggesting that both regions influenced GY performance under water-limited conditions. *GRMZM2G173084* colocalised in this interval, which indicated its promising role in drought tolerance. *Zm00001d044411* for EBM-WS-18 fell in a QTL hotspot in Chr. 3: 219.8–223.7 Mb for GY, ESP, and ASI [39], and its extended region harbours several QTLs responsible for stay-green, leaf senescence, and chlorophyll content identified under normal growth and water stress conditions in maize [29,40,42,43]. *Zm00001d032084* (chaperone protein dnaJ) for ASI-Delay-18 and *Zm00001d026286* (40S ribosomal protein S11) for ASI-WS-18 colocalised in QTL clusters in bin 1.07 and 10.05–10.07, respectively, each harbouring at least three maize-flowering-time QTLs under different planting densities [44]. *Zm00001d024783* (BHLH transcriptional factor 117) for PH-WW-18 and *Zm00001d003939* (11-ß-hydroxysteroid dehydrogenase) for PH-WW-17 were localised in QTL (bin 10.07 and bin 2.05–2.07) for plant height of forage maize [45]. These results indicated that these colocalised genes might play important roles in maize development under different conditions, though further functional validations are needed.

Members of zinc-finger family protein play critical roles in plant growth and developmental processes, including flowering, senescence, and also abiotic stress responses [46,47]. A C_2_H_2_ zinc-finger transcription factor determines stomatal closure by modulating H_2_O_2_-homeostasis related genes, for example, *peroxidases*, *glutathione S-transferase*, and *cytochrome P450s* [48]. Hence, drought response in rice is regulated. *Zm00001d042997* was identified encoding a HIT-type zinc-finger family protein and was associated with ASI-WW and ASI-WS, indicating a potential conserved abiotic-stress-tolerant role of zinc-finger family protein. It was stated that the F-box domain-containing protein ARABIDILLO-1 is conserved in plants, involved in root architecture development, and functions during rice abiotic stress, mainly through regulating root branching and lateral root development [49,50]. It was reported that ARABIDILLO-1-mediated protein degradation, most likely through modulating the GA3 signalling pathway [49]. However, another study revealed that plants with ARABIDILLO-1 knockout and overexpression responded normally to auxin and abscisic acid [51]. *Zm00001d029938*, encoding ARABIDILLO-1 in maize, was associated with ASI in this study. However, whether it functions in maize drought tolerance was not clearly determined. For EBM, an SNP (S5_27121944), which is annotated as pyridoxal phosphate-dependent transferase (Zm00001d013992), was consistently associated for consecutive two years under drought regime (Table 3) and was upregulated by drought stress. The lead SNP S5_27121944 (A/G) in *Zm00001d013992* separated the association panel into two groups, and only around 6% lines (including BY855 and BY4960, etc) carried the favourable haplotype, exhibiting higher ear biomass, 3 g under WS (Figure 7A). Pyridoxal phosphate (PLP) is an active form of pyridoxine (vitamin B6), which functions as a coenzyme in several reactions such as decarboxylation, deamination, and transamination. The PLP dependent enzymes mainly perform in amino acid biosynthesis and the metabolism of its derived metabolites. Therefore, it is interesting to speculate that Zm00001d013992 might be involved in amino acid metabolism and promotes ear development in maize under drought stress conditions. Potential causal SNPs of candidate drought-tolerant genes could be used for drought-tolerant maize improvement through both genome selection and genome editing.

## 5. Conclusions

The findings of this study provide insights into the genetic basis of drought tolerance at the flowering stage, especially for the female inflorescence’s development. The overlapping genes are proposed as candidate genes for drought tolerance in maize. Moreover, those lines carrying favourable alleles could be used for drought-tolerance marker development, which is of benefit for future marker-assisted or genome-wide selection for drought-tolerant maize breeding. Future investigation is needed to explore the candidate gene function using CRISPR/Cas9 mediated genome editing and the underlying molecular mechanism of maize ear and silk development under water deficit condition.

## Figures and Tables

**Table 1:** Descriptive statistics and heritability estimate for the traits of association panel.

Traits	Range	Mean ± SD	CV (%)	(*H*^2^) %	*p* Value
ASI-WS	1.67–21.42	5.58 ± 2.39	42.97	89.31	2.6 × 10^−3^
ASI-WW	0.56–18.15	4.43 ± 2.17	48.89	87.45	
EBM-WS	0.48–3.08	1.42 ± 0.41	29.15	86.78	3.2 × 10^−5^
EBM-WW	0.48–3.81	1.71 ± 0.47	27.20	86.64	
PH-WS	43.21–169.72	111.36 ± 21.54	19.34	92.52	2.7 × 10^−4^
PH-WW	51.23–183.74	127.22 ± 21.53	16.92	94.60	

ASI, anthesis-silking interval, days; EBM, ear biomass, g; PH, plant height, cm; SD, standard deviation; CV, coefficient of variance; *H*^2^, Broad-sense heritability.

**Table 2:** Correlation analysis based on BLUP values across three-year environments under drought (WS, above diagonal) and well-watered (WW, under diagonal) regimes.

Trait	ASI	EBM	PH
ASI		−0.09	0.13 *
EBM	−0.04		0.22 **
PH	0.15 *	0.18 **	

ASI, anthesis-silking interval; EBM, ear biomass; PH, plant height; *, ** significant at *P* < 0.05, 0.01, respectively.

**Table 3:** Annotation of SNPs associated with ASI, EBM, and PH under multiple environments.

Traits	Marker	Chr.	Position	*p* Value	*R* ^2^	Gene ID	Annotation
ASI-WS-18	S1_93513564	1	93513564	5.38 × 10^−6^	0.08207	*Zm00001d029938*	Protein ARABIDILLO 1
	S1_93277641	1	93277641	6.06 × 10^−6^	0.08113	*Zm00001d029937*	Glycoprotein
	PZE-103003226	3	2449913	1.03 × 10^−7^	0.14322	*Zm00001d039319*	Tic22-like family protein
	chr3.S_183263192	3	183319292	1.01 × 10^−5^	0.07963	*Zm00001d042997*	HIT-type zinc-finger family protein
ASI-WW-18	S1_93277641	1	93277641	2.20 × 10^−7^	0.1079	*Zm00001d029937*	Glycoprotein
	S1_93277775	1	93277775	3.28 × 10^−7^	0.10511		
	S1_93278150	1	93278150	7.29 × 10^−7^	0.09852		
	S1_93513564	1	93513564	1.01 × 10^−6^	0.09549	*Zm00001d029938*	Protein ARABIDILLO 1
	S1_93507046	1	93507046	2.48 × 10^−6^	0.08831		
	S1_93505855	1	93505855	3.76 × 10^−6^	0.08489		
	S1_93509892	1	93509892	3.76 × 10^−6^	0.08489		
	S1_93510646	1	93510646	3.76 × 10^−6^	0.08489		
	S1_93511155	1	93511155	3.76 × 10^−6^	0.08489		
	S1_93510058	1	93510058	8.64 × 10^−6^	0.07831		
	S1_93511521	1	93511521	8.64 × 10^−6^	0.07831		
	S1_93513096	1	93513096	8.64 × 10^−6^	0.07831		
	PZE-103003226	3	2449913	1.64 × 10^−6^	0.10835	*Zm00001d039319*	Tic22-like family protein
	chr3.S_183263192	3	183319292	1.66 × 10^−6^	0.09449	*Zm00001d042997*	HIT-type zinc-finger family protein
	S3_183315457	3	183315457	1.91 × 10^−5^	0.09027		
	S3_183315658	3	183315658	1.91 × 10^−6^	0.09027		
	S3_183316916	3	183316916	1.91 × 10^−6^	0.09027		
	S3_183318642	3	183318642	1.91 × 10^−6^	0.09027		
	S3_183315400	3	183315400	5.78 × 10^−6^	0.08148		
	S3_183311733	3	183311733	7.14 × 10^−6^	0.07982		
	S3_183311777	3	183311777	7.14 × 10^−6^	0.07982		
EBM-WS-17	chr7.S_116288756	7	116316709	5.92 × 10^−6^	0.1034	*Zm00001d020506*	26S proteasome non-ATPase regulatory subunit 9
	chr7.S_116288791	7	116316744	5.92 × 10^−6^	0.1034		
	chr7.S_116288792	7	116316745	5.92 × 10^−6^	0.1034		
	chr7.S_116285652	7	116313605	1.01 × 10^−5^	0.09798		
	chr7.S_116285655	7	116313608	1.01 × 10^−5^	0.09798		
EBM-WW-17	S7_116315576	7	116315576	1.17 × 10^−6^	0.11793	*Zm00001d020506*	26S proteasome non-ATPase regulatory subunit 9
	S7_116316425	7	116316425	1.17 × 10^−6^	0.11793		
	S7_116316559	7	116316559	1.17 × 10^−6^	0.11793		
	chr7.S_116288756	7	116316709	1.29 × 10^−6^	0.12102		
	chr7.S_116288791	7	116316744	1.29 × 10^−6^	0.12102		
	chr7.S_116288792	7	116316745	1.29 × 10^−6^	0.12102		
	chr7.S_116285652	7	116313605	3.42 × 10^−6^	0.11084		
	chr7.S_116285655	7	116313608	3.42 × 10^−6^	0.11084		
	S7_116314423	7	116314423	1.81 × 10^−6^	0.11403		
	S7_116316667	7	116316667	2.11 × 10^−6^	0.11193		
EBM-WS-18	S5_27121944	5	27121944	3.25 × 10^−6^	0.08944	*Zm00001d013992*	Pyridoxal phosphate-dependent transferase family protein
EBM-WS-20	S5_27121944	5	27121944	9.15 × 10^−6^	0.09491	*Zm00001d013992*	Pyridoxal phosphate-dependent transferase family protein
PH-WS-17	chr2.S_68691618	2	69321921	2.98 × 10^−7^	0.14098	*Zm00001d003939*	11-ß-hydroxysteroid dehydrogenase
	chr2.S_68691621	2	69321924	2.98 × 10^−7^	0.14098		
	S2_218026770	2	218026770	1.11 × 10^−6^	0.11601	*Zm00001d007189*	Uncharacterised
	S2_226449870	2	226449870	2.08 × 10^−6^	0.10972	*GRMZM2G070937*	Leu-rich repeat protein kinase family protein
PH-WW-17	chr2.S_68691618	2	69321921	7.15 × 10^−8^	0.15528	*Zm00001d003939*	11-ß-hydroxysteroid dehydrogenase
	chr2.S_68691621	2	69321924	7.15 × 10^−8^	0.15528		
	S2_218026770	2	218026770	1.95 × 10^−6^	0.11069	*Zm00001d007189*	Uncharacterised
	S2_226449870	2	226449870	9.07 × 10^−6^	0.09541	*GRMZM2G070937*	Leu-rich repeat protein kinase family protein
PH-WS-18	S8_163927011	8	163927011	4.47 × 10^−6^	0.07836	*Zm00001d012167*	Silk fibroin (SF16) protein
PH-WW-18	S8_163927011	8	163927011	8.67 × 10^−6^	0.07275	*Zm00001d012167*	Silk fibroin (SF16) protein
	S8_163927012	8	163927012	9.66 × 10^−6^	0.07196		

## Data Availability

GWAS results are provided in supplementary data, and the genotypic data of the association panel are available on request from the corresponding author.

## Supplementary Materials

The following information is available online at https://www.mdpi.com/article/10.3390/genes13040564/s1, Table S1: List of inbred lines used for GWAS analysis, Table S2: Annotation of SNPs associated with ASI under two water regimes, Table S3: Annotation of SNPs associated with ear biomass at silking date (EBM) under two water regimes, Table S4: Annotation of SNPs associated with plant height under two water regimes, Table S5: Haplotype analysis of *Zm00001d013992* for EBM in 2018 and 2020, Table S6: Haplotype analysis of *Zm00001d020506* for EBM in 2017, Table S7: Haplotype analysis of *Zm00001d029937* for ASI in 2018, Table S8: Haplotype analysis of *Zm00001d029938* for ASI in 2018, Table S9: Haplotype analysis of *Zm00001d039319* for ASI in 2018, Table S10: Haplotype analysis for *Zm00001d042997* for ASI in 2018.
