# Supplementary material for: Dissection of Maize Drought Tolerance at the Flowering Stage Using Genome-Wide Association Studies
Source: Genes (Basel). 2022 Mar 23;13(4):564. doi: 10.3390/genes13040564 (PMC9031386; doi:10.3390/genes13040564)
Supplement: Supplementary file 1 [file genes-13-00564-s001.zip › genes-1657528-supplementary.pdf]

Table S1 List of inbred lines used for GWAS analysis

No.	Line	No.	Line	No.	Line	No.	Line	No.	Line
1	CML172	57	CIMBL91	113	CIMBL53	169	GEMS11	225	LK11
2	LIAO5262	58	CIMBL51	114	CIMBL3	170	TY11	226	150
3	CML189	59	CIMBL19	115	CIMBL156	171	GEMS49	227	ZHENG28
4	CIMBL2	60	B11	116	CML20	172	GL178	228	TY3
5	CIMBL55	61	CIMBL13	117	CIMBL75	173	QI319	229	GEMS42
6	CML423	62	GEMS13	118	CIMBL82	174	SW92E114	230	CIMBL147
7	CIMBL125	63	CIMBL88	119	JIAO51	175	CIMBL111	231	CIMBL140
8	GEMS23	64	CIMBL43	120	GEMS14	176	CIMBL66	232	CA47
9	CIMBL122	65	CIMBL40	121	LX9801	177	DAN340	233	GEMS39
10	CML426	66	CIMBL79	122	CIMBL6	178	CML139	234	CIMBL116
11	CIMBL38	67	CIMBL150	123	BY855	179	CIMBL10	235	GEMS3
12	CML290	68	CIMBL115	124	SHEN5003	180	CIMBL54	236	CML162
13	CIMBL123	69	CIMBL81	125	CIMBL96	181	ZHENG30	237	CIMBL15
14	CIMBL102	70	CIMBL113	126	CIMBL93	182	268	238	DAN4245
15	CML171	71	CIMBL70	127	CIMBL69	183	TY1	239	CIMBL16
16	CML304	72	CIMBL92	128	07KS4	184	CIMBL5	240	CIMBL133
17	CIMBL77	73	CIMBL105	129	LG001	185	GEMS16	241	CML361
18	CIMBL124	74	CML479	130	Chang7-2	186	835A	242	CIMBL129
19	CIMBL120	75	CIMBL22	131	RY713	187	GEMS36	243	TY6
20	CML69	76	CIMBL32	132	GEMS35	188	TY4	244	CHENG698
21	CIMBL52	77	GEMS32	133	GEMS66	189	GEMS5	245	BY815
22	CML32	78	CML31	134	GEMS28	190	CML170	246	CML323
23	CML493	79	CIMBL106	135	GY386	191	CIMBL62	247	GEMS33
24	CIMBL86	80	CIMBL18	136	JH96C	192	CIMBL119	248	CML121

25	CIMBL48	81	CIMBL74	137	GEMS31	193	CML130	249	CIMBL139
26	CIMBL121	82	CML338	138	BY813	194	GEMS20	250	CIMBL98
27	CML163	83	CIMBL108	139	CIMBL49	195	B151	251	TIAN77
28	CIMBL157	84	CML192	140	GEMS37	196	CIMBL50	252	GEMS17
29	CIMBL68	85	CML454	141	7327	197	GEMS40	253	GEMS15
30	CML496	86	CIMBL127	142	CIMBL60	198	SY1032	254	TY5
31	CIMBL114	87	CML165	143	CML191	199	GEMS50	255	CML122
32	CIMBL152	88	CIMBL25	144	CML118	200	DH3732	256	05WN230
33	CIMBL29	89	CIMBL28	145	DAN599	201	CML411	257	GEMS6
34	GEMS21	90	CIMBL11	146	BY4960	202	CIMBL23	258	BZN
35	ZHONG69	91	CIMBL7	147	D863F	203	CIMBL142	259	ZAC546
36	GEMS2	92	BY4944	148	K22	204	GEMS29	260	DONG46
37	DAN3130	93	HZ4	149	DAN360	205	GEMS62	261	DONG237
38	CHANG3	94	YE515	150	B77	206	GEMS54	262	WH413
39	Q319-Bc6	95	BY4839	151	647	207	LY042	263	GEMS61
40	K10	96	CIMBL145	152	CML114	208	M153	264	TY-HZ4
41	GEMS55	97	CIMBL59	153	CIMBL17	209	MO17	265	335F
42	MO113	98	B113	154	CIMBL153	210	GY1007	266	335M
43	7884-4HT	99	Chuan48-2	155	CML116	211	WU109	267	YE8001
44	GEMS63	100	M97	156	CIMBL144	212	5213	268	ZH68
45	J4112	101	JH59	157	CIMBL42	213	ZHENG653	269	GY462
46	CML325	102	YE478	158	LV28	214	CIMBL141	270	478-BC6
47	LIAO159	103	CF773-2	159	EN25	215	P178	271	ZB648
48	HYS	104	Y2348	160	Z2018F	216	SY1039	272	GEMS58
49	ZHENG58	105	GEMS59	161	975-12	217	ZHENG29	273	CIMBL95
50	GEMS64	106	SY1052	162	4F1	218	LIAO5114	274	CIMBL83

51	GEMS44	107	JY01	163	TY2	219	GEMS51	275	SI273
52	JI853	108	CIMBL1	164	ZZ01	220	Y1382	276	4019
53	GEMS1	109	1462	165	ZHI41	221	JI63	277	YE52106
54	9642	110	FCD0602	166	B110	222	835B	278	QI205
55	526018	111	BY809	167	L3180	223	CIMBL89	279	P138
56	RY729	112	GEMS18	168	GEMS9	224	BY807	-	-

Table S2. Annotation of SNPs associated with ASI under two water regimes

Traits	Marker	Chr	Position	**P value**	R^2	Gene ID	Annotation
ASI-WW-17	S2_196363855	2	196363855	8.08×10^{-6}	0.09659	*Zm00001d006212*	Pseudo-response regulator homolog 1
	S4_167414078	4	167414078	6.39×10^{-6}	0.09828	*Zm00001d051812*	Histidine kinase
	S4_167413941	4	167413941	1.01×10^{-5}	0.0939		
	S8_167328471	8	167328471	2.50×10^{-6}	0.10746	*Zm00001d012361*	Ataxin-3
	chr8.S_141931981	8	141466190	4.28×10^{-6}	0.10757	*Zm00001d011298*	C3HC zinc finger-like family protein
	S9_147770955	9	147770955	1.61×10^{-6}	0.11178	*Zm00001d048129*	Uncharacterized
	S9_147771006	9	147771006	5.37×10^{-6}	0.09997		
ASI-WW-17	chr2.S_235348089	2	236172843	8.63×10^{-6}	0.10309	*Zm00001d007934*	U3 SnRNA-associated protein 6
	S3_229071054	3	229071054	6.23×10^{-6}	0.10226	*Zm00001d044593*	Nuclear pore complex protein NUP98A
	S7_134912267	7	134912267	6.85×10^{-7}	0.12185	*Zm00001d020983*	30S ribosomal protein S16-like
	S7_134881654	7	134881654	3.37×10^{-7}	0.12714	*GRMZM2G173084*	Uncharacterized
	S7_134880687	7	134880687	8.44×10^{-7}	0.12031		
	PZE-107079981	7	134881823	1.04×10^{-6}	0.12823		
	S7_134880722	7	134880722	1.31×10^{-6}	0.11362		
	S7_134877422	7	134877422	2.47×10^{-6}	0.10743		
ASI-Delay-17	S3_199628962	3	199628962	1.14×10^{-5}	0.09626	*Zm00001d043519*	Uncharacterized
	chr8.S_166831645	8	166381545	1.05×10^{-5}	0.09997	*Zm00001d012327*	Uncharacterized
ASI-WS-18	S1_227596184	1	227596184	5.92×10^{-6}	0.08128	*GRMZM5G814683*	dof13-C2C2-Dof-transcription factor
	chr1.S_227534674	1	227596136	7.85×10^{-6}	0.08164		
	chr1.S_227535817	1	227597279	7.88×10^{-6}	0.08161		
	S10_141493232	10	141493232	4.52×10^{-6}	0.08344	*Zm00001d026286*	40S ribosomal protein S11
	chr10.S_65474329	10	65487087	7.86×10^{-6}	0.08164	*Zm00001d024325*	probable protein phosphatase 2C 44
	S1_93513564	1	93513564	5.38×10^{-6}	0.08207	*Zm00001d029938*	Protein ARABIDILLO 1
	S1_93277641	1	93277641	6.06×10^{-6}	0.08113	*Zm00001d029937*	Glycoprotein
	PZE-103003226	3	2449913	1.03×10^{-6}	0.14322	*Zm00001d039319*	Tic22-like family protein
	chr3.S_183263192	3	183319292	1.01×10^{-5}	0.07963	*Zm00001d042997*	HIT-type Zinc finger family protein
ASI-WW-18	S1_93277641	1	93277641	2.20×10^{-7}	0.1079	*Zm00001d029937*	Glycoprotein
	S1_93277775	1	93277775	3.28×10^{-7}	0.10511		
	S1_93278150	1	93278150	7.29×10^{-7}	0.09852		
	S1_93513564	1	93513564	1.01×10^{-6}	0.09549	*Zm00001d029938*	Protein ARABIDILLO 1
	S1_93507046	1	93507046	2.48×10^{-6}	0.08831		
	S1_93505855	1	93505855	3.76×10^{-6}	0.08489		
	S1_93509892	1	93509892	3.76×10^{-6}	0.08489		
	S1_93510646	1	93510646	3.76×10^{-6}	0.08489		
	S1_93511155	1	93511155	3.76×10^{-6}	0.08489		
	S1_93510058	1	93510058	8.64×10^{-6}	0.07831		
	S1_93511521	1	93511521	8.64×10^{-6}	0.07831		

	S1_93513096	1	93513096	8.64×10^{-6}	0.07831		
	PZE-103003226	3	2449913	1.64×10^{-6}	0.10835	*Zm00001d039319*	Tic22-like family protein
	chr3.S_183263192	3	183319292	1.66×10^{-6}	0.09449	*Zm00001d042997*	HIT-type Zinc finger family protein
	S3_183315457	3	183315457	1.91×10^{-6}	0.09027		
	S3_183315658	3	183315658	1.91×10^{-6}	0.09027		
	S3_183316916	3	183316916	1.91×10^{-6}	0.09027		
	S3_183318642	3	183318642	1.91×10^{-6}	0.09027		
	S3_183315400	3	183315400	5.78×10^{-6}	0.08148		
	S3_183311733	3	183311733	7.14×10^{-6}	0.07982		
	S3_183311777	3	183311777	7.14×10^{-6}	0.07982		
	S2_7412314	2	7412314	7.30×10^{-6}	0.07974	*GRMZM2G506383*	Uncharacterized
	S2_23597795	2	23597795	9.79×10^{-6}	0.08587	*Zm00001d002837*	Uncharacterized
	S3_183312336	3	183312336	4.81×10^{-7}	0.10139	*Zm00001d042996*	2-oxoglutarate (2OG)
	PZE-103125662	3	183312373	9.90×10^{-7}	0.10227		
	S3_183312126	3	183312126	1.80×10^{-6}	0.09076		
	S3_183313223	3	183313223	2.45×10^{-6}	0.08831		
	S3_183312628	3	183312628	2.50×10^{-6}	0.08812		
	S3_183312177	3	183312177	3.32×10^{-6}	0.08588		
	S3_183311690	3	183311690	5.08×10^{-6}	0.08568		
	S3_183313231	3	183313231	5.29×10^{-6}	0.08311		
	S3_183321823	3	183321823	1.91×10^{-6}	0.09027	*GRMZM2G037727*	Uncharacterized
	S3_183321994	3	183321994	1.91×10^{-6}	0.09027		
	S3_183322659	3	183322659	1.91×10^{-6}	0.09027	*GRMZM2G033058*	ATP binding cassette protein 1
	chr3.S_183268090	3	183324190	6.33×10^{-6}	0.08345		
	chr8.S_145191985	8	144727028	8.76×10^{-6}	0.0808	*Zm00001d011398*	MAKR gene family protein
	S8_64083558	8	64083558	9.81×10^{-6}	0.07731	*Zm00001d009446*	VIP1 protein (bZIP TF in arabidopsis)
	S9_155724571	9	155724571	1.00×10^{-6}	0.09546	*Zm00001d048551*	cnr6 - Cell Number Regulator 6
	S9_155728197	9	155728197	9.69×10^{-6}	0.0804	*Zm00001d048552*	Helicase CHR10
	S10_120515824	10	120515824	6.74×10^{-6}	0.08027	*Zm00001d025533*	Oxidoreductase, aldo/keto reductase
ASI- Delay-18	S1_209173685	1	209173685	2.57×10^{-6}	0.09138	*Zm00001d032084*	Chaperone protein dnaJ
	PZE-101166186	1	209173942	1.02×10^{-5}	0.08636		
	S8_174647623	8	174647623	9.49×10^{-6}	0.08062	*Zm00001d012766*	Probable pectin-esterase 53
ASI-WS-20	S1_260576586	1	260576586	9.15×10^{-6}	0.09576	*Zm00001d033510*	Uncharacterized
ASI-WW-20	chr5.S_182249972	5	182300531	4.68×10^{-6}	0.11738	*Zm00001d017146*	Uncharacterized

Table S3. Annotation of SNPs associated with ear biomass at silking date (EBM) under two water regimes

**Traits**	**Marker**	**Chr**	**Position**	***P* value**	**R^2**	**Gene ID**	**Annotation**
EBM-WS-17	S1_175670732	1	175670732	7.31×10^{-6}	0.09788	*Zm00001d031109*	yab11 - C2C2-YABBY-TF 11
	S5_56454527	5	56454527	4.87×10^{-6}	0.1018	*Zm00001d014669*	Cadmium/zinc-transporting ATPase
	S8_161338291	8	161338291	8.61×10^{-7}	0.12174	*Zm00001d012031*	Uncharacterized
	S8_161329625	8	161329625	8.02×10^{-6}	0.09693	*Zm00001d012030*	E2 protein isoform 5
EBM-WW-17	S7_116404702	7	116404702	1.62×10^{-6}	0.11426	*Zm00001d020511*	Ubiquitin-NEDD8-like protein RUB1
	S7_116402724	7	116402724	1.06×10^{-6}	0.11891	*Zm00001d020510*	COX17-Cytochrome C oxidase copper chaperone
	S7_116399400	7	116399400	1.42×10^{-6}	0.11558		
	S7_116399515	7	116399515	1.74×10^{-6}	0.11357		
	S7_116399159	7	116399159	2.11×10^{-6}	0.11193		
	S7_116399213	7	116399213	2.11×10^{-6}	0.11193		
	S7_116402441	7	116402441	2.11×10^{-6}	0.11193		
	S7_116402616	7	116402616	3.09×10^{-6}	0.11297		
	S7_116402618	7	116402618	3.09×10^{-6}	0.11297		
	S7_116402494	7	116402494	3.11×10^{-6}	0.10932		
	chr7.S_116374791	7	116402744	3.44×10^{-6}	0.11075		
	chr7.S_116371117	7	116399070	4.01×10^{-6}	0.10917		
	S8_133600163	8	133600163	4.83×10^{-6}	0.10334	*GRMZM2G077691*	Transposable element
EBM-ratio-17	S5_48601401	5	48601401	7.77×10^{-6}	0.09738	*GRMZM5G848124*	Uncharacterized
	chr5.S_160451114	5	160490546	1.00×10^{-5}	0.09881	*Zm00001d016479*	Importin beta-like SAD2
EBM-WS-18	S2_171411900	2	171411900	7.54×10^{-7}	0.10143	*Zm00001d005478*	Cellulose synthase A catalytic subunit 6
	S2_214078538	2	214078538	7.88×10^{-7}	0.10137	*Zm00001d007009*	DNAJ heat shock protein
	S2_214059088	2	214059088	1.35×10^{-6}	0.09632		
	S2_214060345	2	214060345	5.53×10^{-6}	0.08468		
	S2_214064988	2	214064988	5.53×10^{-6}	0.08468		
	S2_214065975	2	214065975	5.53×10^{-6}	0.08468		
	S2_214058926	2	214058926	6.61×10^{-6}	0.08338		
	S2_219686839	2	219686839	9.95×10^{-6}	0.0799	*GRMZM2G169681*	Uncharacterized

	chr3.S_223690139	3	223776896	5.17×10^{-6}	0.0872	*Zm00001d044411*	Putative calcium-binding protein CML15
	S3_224664363	3	224664363	8.95×10^{-6}	0.08207	*GRMZM2G034430*	Carbon catabolite repressor protein4 homolog6
	S3_223834589	3	223834589	8.99×10^{-6}	0.08084	*Zm00001d044418*	Uncharacterized
	S5_33998541	5	33998541	8.38×10^{-6}	0.08281	*Zm00001d014180*	Flowering locus K homology domain
	chr6.S_22666648	6	22666161	4.05×10^{-6}	0.08926	*Zm00001d035318*	Uncharacterized
	chr6.S_22666649	6	22666162	4.05×10^{-6}	0.08926		
	chr6.S_22666666	6	22666179	4.05×10^{-6}	0.08926		
	chr6.S_22666667	6	22666180	8.98×10^{-6}	0.08257		
	S7_140295502	7	140295502	1.47×10^{-6}	0.09638	*GRMZM2G176403*	Uncharacterized
	chr7.S_138909741	7	138946464	7.95×10^{-6}	0.0836	*Zm00001d021136*	Salt-inducible protein ser/thr/tyr kinase
	chr7.S_130785502	7	130818260	8.27×10^{-6}	0.08326	*Zm00001d020853*	RNA 3-terminal phosphate cyclase-like
	chr7.S_130785503	7	130818261	8.27×10^{-6}	0.08326		
	chr7.S_130785504	7	130818262	8.27×10^{-6}	0.08326		
	chr7.S_130785505	7	130818263	8.27×10^{-6}	0.08326		
	S9_153782708	9	153782708	2.84×10^{-6}	0.09024	*Zm00001d048454*	fha17 - FHA-transcription factor 17
EBM-WW-18	chr1.S_288855240	1	288926160	3.11×10^{-7}	0.11155	*Zm00001d034485*	Uncharacterized
	chr1.S_288855249	1	288926169	3.11×10^{-7}	0.11155		
	S1_288927107	1	288927107	8.34×10^{-7}	0.10095	*Zm00001d034486*	Small GTP-binding protein domain
	chr1.S_220201354	1	220253493	8.65×10^{-7}	0.1027	*Zm00001d032350*	Uncharacterized
	chr1.S_220205446	1	220257585	2.62×10^{-6}	0.09324		
	S1_220254047	1	220254047	3.07×10^{-6}	0.09008		
	S1_220253843	1	220253843	3.36×10^{-6}	0.08949		
	S1_220254311	1	220254311	3.36×10^{-6}	0.08949		
	S1_220254828	1	220254828	3.36×10^{-6}	0.08949		
	S1_220255630	1	220255630	3.36×10^{-6}	0.08949		
	S1_220255662	1	220255662	3.36×10^{-6}	0.08949		
	S1_220256130	1	220256130	3.36×10^{-6}	0.08949		
	S1_220256256	1	220256256	5.44×10^{-6}	0.08551		
	chr1.S_93801342	1	93810262	7.62×10^{-6}	0.08422	*Zm00001d029946*	Uncharacterized

chr1.S_93801339	1	93810259	9.36×10^{-6}	0.0825		
chr1.S_93801340	1	93810260	9.36×10^{-6}	0.0825		
S1_248346174	1	248346174	8.55×10^{-6}	0.08163	*Zm00001d033152*	ent-cassadiene C2-hydroxylase like
S1_291598670	1	291598670	8.97×10^{-6}	0.08124	*Zm00001d034581*	Uncharacterized
S2_3377419	2	3377419	2.62×10^{-6}	0.09146	*GRMZM2G047590*	Peptidyl-prolyl cis-trans isomerase
S2_157500038	2	157500038	5.32×10^{-6}	0.08577	*Zm00001d005156*	Uncharacterized
S2_213196915	2	213196915	5.57×10^{-6}	0.08522	*Zm00001d006917*	Leucine-rich repeat protein kinase protein
chr3.S_68601919	3	68605119	4.38×10^{-7}	0.10856	*GRMZM2G132222*	Uncharacterized
S3_13321362	3	13321362	2.92×10^{-6}	0.0905	*Zm00001d039703*	Autophagy-related protein 18c
S3_197351667	3	197351667	4.56×10^{-6}	0.08695	*Zm00001d043444*	Uncharacterized
S3_173846291	3	173846291	8.07×10^{-6}	0.08777	*Zm00001d042673*	Amido-phospho-ribosyl-transferase
S4_202697624	4	202697624	7.94×10^{-8}	0.12095	*Zm00001d052974*	Uncharacterized
chr4.S_202441033	4	202618874	1.09×10^{-7}	0.1207	*GRMZM5G866910*	Uncharacterized
chr4.S_202441044	4	202618885	1.09×10^{-7}	0.1207		
chr4.S_202440988	4	202618829	1.88×10^{-6}	0.09604		
chr4.S_202440997	4	202618838	1.88×10^{-6}	0.09604		
chr4.S_202440908	4	202618749	2.49×10^{-6}	0.09366		
chr4.S_202440922	4	202618763	2.49×10^{-6}	0.09366		
S4_2116917	4	2116917	6.42×10^{-6}	0.08461	*Zm00001d048655*	Splicing factor U2AF subunit
S4_199975624	4	199975624	7.68×10^{-6}	0.08251	*Zm00001d052903*	3-hydroxy-3-methylglutaryl-coenzyme A reductase
S4_199977361	4	199977361	7.68×10^{-6}	0.08251		
S4_224578178	4	224578178	7.82×10^{-6}	0.08237	*Zm00001d053376*	Ankyrin repeat family protein
S5_202476687	5	202476687	7.24×10^{-6}	0.12174	*Zm00001d017826*	3-hydroxy-3methylglutaryl-coenzyme A reductase1
S5_202472440	5	202472440	2.80×10^{-7}	0.11018		
S5_202473422	5	202473422	7.75×10^{-6}	0.08247		
S5_202473991	5	202473991	7.75×10^{-6}	0.08247		
S5_215101922	5	215101922	8.02×10^{-6}	0.08221	*Zm00001d018443*	Ankyrin repeat domain-containing protein
S6_84691343	6	84691343	2.42×10^{-6}	0.09206	*Zm00001d036418*	Uncharacterized
chr6.S_60854926	6	61014126	2.43×10^{-6}	0.09387	*GRMZM2G083408*	Uncharacterized

	chr6.S_60854810	6	61014010	3.91×10^{-6}	0.08983		
	S8_170767138	8	170767138	3.70×10^{-6}	0.08853	*Zm00001d012550*	Brassinosteroid Insensitive 1-associated kinase1
	chr8.S_157000495	8	156540497	3.82×10^{-6}	0.09003	*Zm00001d011805*	PH, RCC1 and FYVE domains-containing protein1
	S9_111272103	9	111272103	2.73×10^{-6}	0.09105	*Zm00001d046981*	Glk32: MYB DNA-binding domain family protein
	S9_111273022	9	111273022	7.44×10^{-6}	0.08277		
	chr10.S_77114987	10	77128337	1.01×10^{-6}	0.10138	*GRMZM2G171236*	Mitochondrial NADH ubiquinone oxidoreductase
	S10_145607097	10	145607097	2.09×10^{-6}	0.09327	*Zm00001d026489*	G-type lectin S-receptor-like ser/thr-protein
	S10_124087230	10	124087230	9.49×10^{-7}	0.09986	*Zm00001d025653*	Uncharacterized
	S10_124086814	10	124086814	2.85×10^{-6}	0.09068		
	S10_124087099	10	124087099	2.85×10^{-6}	0.09068		
	S10_124088126	10	124088126	2.85×10^{-6}	0.09068		
	S10_13791295	10	13791295	3.34×10^{-6}	0.08937	*Zm00001d023659*	Uncharacterized
	chr10.S_13785048	10	13790345	5.08×10^{-6}	0.08762		
	chr10.S_13785085	10	13790382	5.08×10^{-6}	0.08762		
	S10_13790953	10	13790953	5.43×10^{-6}	0.08617		
	S10_137779511	10	137779511	2.91×10^{-6}	0.09103	*Zm00001d026124*	Ser/Thr-rich protein T10 in DGCR region
	S10_137778405	10	137778405	6.09×10^{-6}	0.08442		
	S10_137779762	10	137779762	6.09×10^{-6}	0.08442		
	chr10.S_16206542	10	16211639	1.01×10^{-5}	0.08185	*Zm00001d023706*	Uncharacterized
EBM-ratio-18	S1_244829817	1	244829817	4.46×10^{-6}	0.08834	*Zm00001d033048*	Protein TIFY 11d
	S1_122479055	1	122479055	8.56×10^{-6}	0.0829	*Zm00001d030348*	Alcohol dehydrogenase superfamily protein
	chr1.S_244834434	1	244893365	9.36×10^{-6}	0.08302	*Zm00001d033049*	Zim motif family protein
	chr3.S_194426127	3	194489685	2.57×10^{-6}	0.09399	*Zm00001d043350*	Indole-3-acetic acid-amido synthetase
	chr3.S_194426048	3	194489606	8.75×10^{-6}	0.08358		
	chr3.S_161574589	3	161617387	4.73×10^{-6}	0.08879	*Zm00001d042361*	Wiscott-Aldrich syndrome, C-terminal
	S3_224976707	3	224976707	6.84×10^{-6}	0.08478	*Zm00001d044457*	Pentatricopeptide repeat-containing protein
	S3_219483425	3	219483425	8.18×10^{-6}	0.08328	*Zm00001d044253*	Putative calmodulin-binding family protein
	S6_34439990	6	34439990	6.46×10^{-6}	0.08528	*Zm00001d035597*	RmlC-like cupins superfamily protein
	chr7.S_157473811	7	157512141	2.63×10^{-6}	0.0938	*GRMZM5G862799*	F-box family protein

	chr10.S_134053634	10	134078101	5.31×10^{-6}	0.0878	*Zm00001d025984*	Glutamic dehydrogenase-2
EBM-WS-20	PZE-101095906	1	94310713	2.03×10^{-6}	0.12344	*GRMZM2G141692*	Uncharacterized
	S4_228551470	4	228551470	6.17×10^{-7}	0.12146	*GRMZM2G092321*	Uncharacterized
	S4_228551398	4	228551398	7.82×10^{-6}	0.09672		
	S6_162532432	6	162532432	8.47×10^{-7}	0.11809	*Zm00001d038892*	Putative clathrin assembly protein
	S6_162534514	6	162534514	8.47×10^{-7}	0.11809		
	S6_162536035	6	162536035	8.47×10^{-7}	0.11809		
	S6_162537399	6	162537399	8.47×10^{-7}	0.11809		
	S6_162533364	6	162533364	7.78×10^{-6}	0.09637		
	S6_162570085	6	162570085	8.47×10^{-7}	0.11809	*Zm00001d038894*	serine/threonine-protein kinase
	S6_162570196	6	162570196	8.47×10^{-7}	0.11809		
	S6_162581385	6	162581385	8.47×10^{-7}	0.11809		
	S7_172922172	7	172922172	5.84×10^{-6}	0.09914	*Zm00001d022451*	CBL-interacting ser/thr protein kinase-11
	S10_137853965	10	137853965	5.53×10^{-6}	0.09998	*Zm00001d026126*	Protein kinase APK1B chloroplast precursor
EBM-WW-20	S3_224575443	3	224575443	7.52×10^{-6}	0.09617	*Zm00001d044443*	ABC transporter G family member 37
	S5_169712325	5	169712325	5.01×10^{-6}	0.10135	*Zm00001d016716*	Integrin beta-1 binding protein2
	S7_145118663	7	145118663	8.77×10^{-6}	0.09469	*Zm00001d021365*	DNA primases
	S8_66507071	8	66507071	4.19×10^{-7}	0.12518	*Zm00001d009508*	DUF1639 family protein
	S8_71150682	8	71150682	4.89×10^{-6}	0.10059	*Zm00001d009603*	Major facilitator super family protein
	S8_68927325	8	68927325	1.00×10^{-5}	0.09339	*Zm00001d009564*	Guanylate-binding family protein
EBM-ratio-20	S1_15169238	1	15169238	9.90×10^{-6}	0.09509	*Zm00001d027856*	Kelch motif family protein
	S7_155627315	7	155627315	6.54×10^{-6}	0.10202	*Zm00001d021707*	Histone H2A
	chr9.S_30644685	9	30657881	7.45×10^{-6}	0.10508	*Zm00001d045644*	Uncharacterized
	chr9.S_30644677	9	30657873	1.02×10^{-5}	0.10181		

Table S4. Annotation of SNPs associated with plant height under two water regimes

**Traits**	**Marker**	**Chr**	**Position**	***P* value**	**R^2**	**Gene ID**	**Annotation**
PH-WS-17	chr2.S_68691618	2	69321921	2.98×10^{-7}	0.14098	*Zm00001d003939*	11-ß-hydroxysteroid dehydrogenase
	chr2.S_68691621	2	69321924	2.98×10^{-7}	0.14098		
	S2_218026770	2	218026770	1.11×10^{-6}	0.11601	*Zm00001d007189*	Uncharacterized
	S2_226449870	2	226449870	2.08×10^{-6}	0.10972	*GRMZM2G070937*	Leu-rich repeat protein kinase family protein
	PZE-102080818	2	65373690	6.66×10^{-6}	0.11358	*Zm00001d003904*	Uncharacterized
	chr2.S_64748927	2	65379330	7.85×10^{-6}	0.1055		
	chr2.S_178974821	2	179624271	1.02×10^{-5}	0.10277	*GRMZM2G030522*	Uncharacterized
PH-WW-17	chr2.S_68691618	2	69321921	7.15×10^{-8}	0.15528	*Zm00001d003939*	11-ß-hydroxysteroid dehydrogenase
	chr2.S_68691621	2	69321924	7.15×10^{-8}	0.15528		
	S2_218026770	2	218026770	1.95×10^{-6}	0.11069	*Zm00001d007189*	Uncharacterized
	S2_226449870	2	226449870	9.07×10^{-6}	0.09541	*GRMZM2G070937*	Leu-rich repeat protein kinase family protein
PH-ratio-17	chr2.S_2082318	2	2084691	8.17×10^{-6}	0.1044	*Zm00001d001865*	Cytokinin response regulator 1
PH-WS-18	S8_163927011	8	163927011	4.47×10^{-6}	0.07836	*Zm00001d012167*	SF16 (Silk Fibroin) protein
PH-WW-18	S8_163927011	8	163927011	8.67×10^{-6}	0.07275	*Zm00001d012167*	SF16 (Silk Fibroin) protein
	S8_163927012	8	163927012	9.66×10^{-6}	0.07196		
	S10_87704955	10	87704955	4.39×10^{-6}	0.07953	*Zm00001d024783*	Bhlh-Transcriptional factor 117
	S10_87705054	10	87705054	9.38×10^{-6}	0.07221		
PH-ratio-18	S7_16920783	7	16920783	9.09×10^{-6}	0.07442	*Zm00001d019114*	rRNA biogenesis protein RRP5
	S9_149312650	9	149312650	7.20×10^{-6}	0.07704	*Zm00001d048194*	hagtf36 - GNAT-TF 36
PH-WS-20	S1_181916365	1	181916365	8.09×10^{-6}	0.10541	*Zm00001d031254*	Non-functional riboflavin biosynthesis protein
PH-WW-20	S8_158798073	8	158798073	6.70×10^{-6}	0.09874	*Zm00001d011891*	Esterase D; S-formylglutathione hydrolase

Table S5. Haplotype analysis of *Zm00001d013992* for EBM in 2018 and 2020

**Haplotype**	**S5_27121944**	**EBM-WS-2018**	**EBM-WS-2020**
HapA	A	1.25	1.42
HapB	G	2.10	2.10

Table S6. Haplotype analysis of *Zm00001d020506* for EBM in 2017

**Haplotype**	**chr7.S_116288756**	**chr7.S_116288791**	**chr7.S_116288792**	**chr7.S_116285652**	**chr7.S_116285655**	**EBM-WS**	**EBM-WW**
HapA	A	A	C	C	T	1.58	1.79
HapB	G	T	T	T	C	1.35	1.55

Table S7. Haplotype analysis of *Zm00001d029937* for ASI in 2018

**Haplotype**	**S1_93277641**	**S1_93277775**	**S1_93278150**	**ASI-WS**	**ASI-WW**
HapA	A	A	C	12.21	11.61
HapB	G	G	G	6.69	5.67

Table S8. Haplotype analysis of *Zm00001d029938* for ASI in 2018

Haplotype	S1_93513564	S1_93507046	S1_93505855	S1_93509892	S1_93510646	S1_93511155	S1_93510058	S1_93511521	S1_93513096	ASI-WS	ASI-WW
HapA	T	C	G	A	T	A	A	T	C	12.63	11.61
HapB	C	T	C	C	C	G	C	C	T	6.64	5.65

Table S9. Haplotype analysis of *Zm00001d039319* for ASI in 2018

**Haplotype**	**PZE-103003226**	**ASI-WS**	**ASI-WW**
HapA	G	5.41	9.70
HapB	T	6.40	11.46

Table S10. Haplotype analysis for *Zm00001d042997* for ASI in 2018

**Haplotype**	**chr3.S_183263192**	**S3_183315457**	**S3_183315658**	**S3_183316916**	**S3_183318642**	**S3_183315400**	**S3_183311733**	**S3_183311777**	**ASI-WS**	**ASI-WW**
HapA	A	A	C	T	C	C	C	G	6.51	5.34
HapB	A	A	C	T	C	T	A	A	6.27	5.73
HapC	T	G	A	C	T	T	A	A	12.15	11.30
